# Breast cancer in lesbians and bisexual women: systematic review of incidence, prevalence and risk studies

**DOI:** 10.1186/1471-2458-13-1127

**Published:** 2013-12-05

**Authors:** Catherine Meads, David Moore

**Affiliations:** 1 Health Economics Research Group, Brunel University, Room 060 Gaskell Building, Uxbridge, Middlesex UB8 3PH, UK; 2 Unit of Public Health, Epidemiology and Biostatistics, University of Birmingham, Birmingham B15 2TT, UK

**Keywords:** Breast cancer, Incidence, Prevalence, Risk, Sexual orientation, Lesbian, Systematic review

## Abstract

**Background:**

The UK Parliamentary Enquiry and USA Institute of Medicine state that lesbians may be at a higher risk of breast cancer but there is insufficient information. Lesbians and bisexual (LB) women have behavioural risk-factors at higher rates compared to heterosexuals such as increased alcohol intake and higher stress levels. Conversely, breast cancer rates are higher in more affluent women yet income levels in LB women are relatively low. This systematic review investigated all evidence on whether there is, or likely to be, higher rates of breast cancer in LB women.

**Methods:**

Cochrane library (CDSR, CENTRAL, HTA, DARE, NHSEED), MEDLINE, EMBASE, PsychINFO, CAB abstracts, Web of Science (SCI, SSCI), SIGLE and Social Care Online databases were searched to October 2013. Unpublished research and specific lesbian, gay and bisexual websites were checked, as were citation lists of relevant papers. Included were studies in LB populations reporting breast cancer incidence or prevalence rates, risk model results or risk-factor estimates. Inclusions, data-extraction and quality assessment were by two reviewers with disagreements resolved by discussion.

**Results:**

Searches found 198 references. No incidence rates were found. Nine studies gave prevalence estimates - two showed higher, four showed no differences, one showed mixed results depending on definitions, one had no comparison group and one gave no sample size. All studies were small with poor methodological and/or reporting quality. One incidence modelling study suggested a higher rate.

Four risk modelling studies were found, one Rosner-Colditz and three Gail models. Three suggested higher and one lower rate in LB compared to heterosexual women. Six risk-factor estimates suggested higher risk and one no difference between LB and heterosexual women.

**Conclusions:**

The only realistic way to establish rates in LB women would be to collect sexual orientation within routine statistics, including cancer registry data, or from large cohort studies.

## Background

Recent US and UK Government policy documents have stated that there may be higher rates of breast cancer in lesbians and bisexual women (LB women), but the evidence that this is based on is unclear. For example, in 2009 the Report by the UK All Party Parliamentary Group on Cancer’s Inquiry into Inequalities in Cancer [1] stated that “Lesbians may be at a higher risk of breast cancer”, and the USA Institute of Medicine’s Report on the Health of Lesbian, Gay, Bisexual, and Transgender People 2011 states that:

“While the relative risk of breast cancer for lesbians and heterosexual women is the topic of much discussion, a definitive answer is still unavailable. It is believed that lesbians may be at higher risk for breast cancer because there is some evidence that they have a higher prevalence of certain risk factors, including nulliparity, alcohol consumption, smoking, and obesity.” [2].

It has long been thought within the LB women’s community that there is a higher rate of breast cancer [3]. As the proportion of LB women in the population is likely to be around 5-7%, increased rates of breast cancer may affect large numbers of LB women. Also, recent research has demonstrated an increased mortality rate from breast cancer in LB women (relative risk 3.2, 95% CI 1.01-10.21) [4]. So if there is an increased risk and an increased mortality rate, this would constitute a considerable health issue for LB women.

However, sexual orientation has not yet become part of demographic data collection for cancer intelligence agencies in the UK or USA nor have any cohort studies measuring incidence of breast cancer collected data on sexual orientation [5]. So health policy is being developed based on lower quality evidence. For example, it may be that the UK policy mentioned above [1] was partly based on an unpublished cross sectional survey conducted by Stonewall charity called the Prescription for Change survey [6]. This collected information from around 6000 LB women using a convenience sample rather than a random population sample. It found a prevalence of breast cancer of 8% in the age group of 40 – 59 years [6]. As the age-specific incidence of breast cancer increases as women get older from 100/100,00 at age 40 to 200/100,00 at age 70 [7], this survey population was relatively young and had an unexpectedly high rate. As the sample size is relatively small the confidence intervals around this point estimate will be large.

There are several reasons as to why there might be an increased risk of breast cancer in lesbians and bisexual (LB) women. For example, there are several known behavioural risk factors at higher rates in LB compared to heterosexual women such as increased alcohol intake and higher stress levels [8]. Also pregnancy rates are likely to be lower. Conversely, breast cancer rates are higher in more affluent women [9] and income levels in UK LB women are known to be relatively low [8]. Also, it is unclear at present as to why lesbians are lesbians but if it is associated with alterations of hormone levels such as oestrogen and progesterone, differential rates of breast cancer could be experienced. An unpublished report into health needs and values of LB women found that cancer was one of the three biggest health concerns for 69% of the sample of 406 women [10].

Considerable work has been undertaken to try to quantify the amount of increased risk in factors associated with an increased risk in breast cancer [11]. For example McPherson et al. [7] listed established and probable risk factors and the relative risks for each factor separately. Incidence risk models are statistical tools that can be used to estimate the probability of an individual of a specific age and set of risk factors developing a condition within a certain time period (such as five years or lifetime). Risk models use characteristics of people or populations which can be environmental, behavioural, genetic or psychological in a statistical model to make a population-based estimate of risk, or to generate an individualised risk estimate. This model then needs to be validated in a second population to ensure that it has reasonably good predictive properties. Frequently the predictive ability of models are not as good in the validation sample as the original sample, so adjustments are made and the model gradually improves. A large number of breast cancer risk models have been developed, the most well-known being the Gail model, but numerous others include the Claus, Tyrer-Cuzick, Jonker and Rosner & Colditz models [12,13].

This systematic review investigates all evidence on whether there is, or likely to be higher rates of breast cancer in LB women. It includes relevant incidence and prevalence studies and all studies where risk factor models or estimates have been applied to any LB population.

## Methods

A protocol was developed and a scoping search was undertaken in November 2009. The following databases were searched: Cochrane library (CDSR, CENTRAL, HTA, DARE, NHSEED), MEDLINE, EMBASE, PsychINFO, CAB abstracts, Web of Science (SCI, SSCI), SIGLE, Social Care Online to February 2010, updated in October 2013. Unpublished research and material only available on lesbian, gay and bisexual (LGB) specific websites was checked. Contact with experts was made, including the Boston Lesbian Health Project (USA). Citation lists of included and excluded studies and reviews were checked. The following search terms were used: lesbian, gay women, queer/bisexual/sexual preference/sexual orientation + female, breast cancer, breast carcinoma, breast neoplasm.

Included were studies in any LB populations measuring and presenting breast cancer incidence or prevalence rates, risk factor estimates or risk model results. All identified citations (titles ± abstracts) were screened by two reviewers. Data extraction and quality assessment were by two reviewers with disagreements resolved through discussion. Quality assessment assessed selection, performance, attrition and detection biases where possible, appropriate to study designs. There are no specific quality assessment checklists for prediction modelling studies but there is a list of criteria recently published by Altman [14]. This was for prognostic rather than predictive studies but the issues are similar. Quality factors relevant to prediction models are listed below.

•Study design – prospective cohort better.

•Patient sample - participants followed up from a common event.

•Sample size – power depends on number of events so if a general population sample needs large numbers or long follow up or both. Suggested needing ten times the number of events to the number of variables studied.

•Incomplete data, missing or lost to follow up are difficult problems that reduce power and probably bias. Completeness of data should be reported.

•Use of continuous variables better than categorisation into low and high risk groups.

•Not useful to have participants split into multiple risk groups, up to three better (low, medium and high risk).

•Needs to have validation study in an independent sample.

Study characteristics and results were tabulated and assessed qualitatively. Results were interpreted in light of methodological strengths and weaknesses identified in quality assessment. No meta-analysis was appropriate because of heterogeneity of study populations and study designs.

## Results

**Figure 1:**
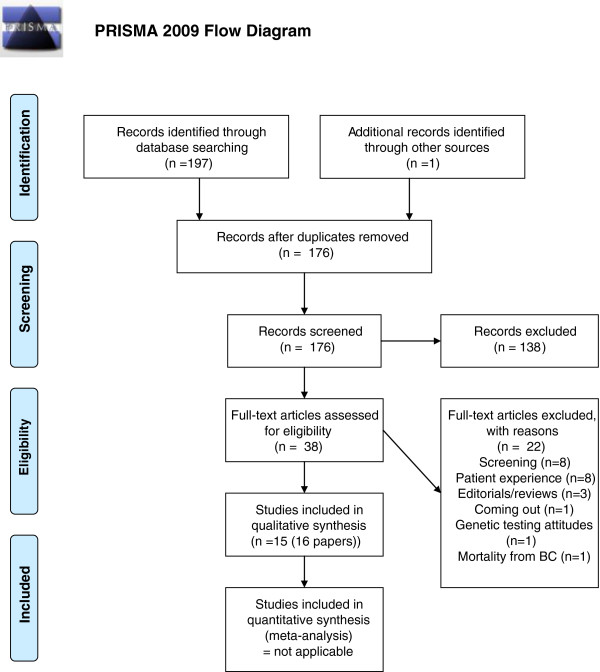
PRISMA diagram.

From the database searches 198 references were found of which 24 were duplicates. Additional internet and citation searches yielded three more references. Flow of review information is shown in Figure 1.

### Incidence and prevalence of breast cancer

There was no published information on incidence of breast cancer in LB women in any country found.

Nine studies were found giving information on prevalence. One was an unpublished cross-sectional survey from the UK (Prescription for Change) [6]. The other eight were published articles from USA [15-21] and from Denmark [22].

Brandenburg et al. (2007) gave a prevalence of 2% lesbian and 3% heterosexual women who had reported ever being diagnosed with breast cancer, but it was not possible to determine the size of the sample these percentages came from and the numbers in the paper did not add up correctly so these results should be treated with caution [15].

Cochran et al. (2001) reported the results of seven large studies on lesbian and bisexual women conducted in various parts of the USA between 1987 and 1996 [16]. The sample sizes varied from 6105 to 322 with a mean of 1484.5 and the ages ranged between 18–75 years. Very little information is available in the paper about how the studies were identified and conducted and how their results were analysed. Six studies provided information on breast cancer history, including one on diagnosis of breast cancer in the previous year. Table 1 is reproduced from this report and shows the age-related distribution of breast cancer prevalence in LB women from the six surveys combined, compared to similar results from the NHANES III study (sample size approximately 9000 women). It can be seen that there is no increase in prevalence. However, the 95% confidence intervals are wide. Unfortunately, the results of the survey that had asked about breast cancer in the previous year were not reported in the journal article and the original report was no longer available, (Personal communication, Anne Pinchak, Houston Lesbian Health Initiative, February 2010).

**Table 1:** **Breast cancer prevalence rates from Cochran et al.**[16]

	**LB women**			NHANES III*	
**Age**	**No**	**% prevalence**	**95% CI**	**% prevalence**	**95% CI**
Under 40	7962	0.2	0.1-0.4	0.2	0.0-0.4
40-49	2671	1.5	0.1-2.5	1.0	0.4-1.9
50-59	739	3.6	2.5-5.3	3.6	0.1-7.0
60-75	182	8.8	5.4-13.9	10.0	3.0-16.9
Combined	11,554	0.9	0.8-1.1	0.9	0.4-1.3

*Standardised by age, ethnicity, education level, geographic region.

Frisch et al. (2003) is a cohort study [22] with linked information on women in registered homosexual partnerships to the Danish cancer registry between 1989 and 1997 using identity-secure data linkage and compared the results for a variety of cancers to that of the entire Danish population. Cancer incidence rates were stratified by sex, age, and 5-year time bands to give expected rates which were then compared to observed rates. The lesbian sample consisted of 1,614 women of whom 161 were aged between 50 and 59, and 58 were aged over 60 years so only 14% of the cohort were over 49 yrs old at time of registration of partnership. The average follow-up period was 4.4 years. Seven women developed breast cancer during the observation period and the relative risk of breast cancer was calculated to be 0.9 (95% CI 0.4-1.9).

It is unclear how representative of the lesbian population this sample was. There is no information on social class or education levels. As 1989 was the year that Denmark passed its law permitting homosexual partnerships (the first in the world), the sample was among the first partnerships to be recognised and many may have been in long-term relationships beforehand. Also, the sample size is relatively small so the 95% confidence intervals for the relative risk estimate are quite wide, and the follow up period is short.

Kavanaugh-Lynch (2002) did not ask sexual orientation directly but used three different demographic indicators to suggest lesbian sexual orientation [17]. These were “no male sexual partners ever” (0.8% of population), “never married and not currently using contraception” (4 – 8%) and “not currently married and using contraception” (9 -12%). It is debatable how accurate these are at indicating lesbians and/ or bisexual women. As the title included “lesbian sexual orientation” this study could be described as misleading. The study found an increased relative risks for breast cancer, with the second group having a statistically significant relative risk of 1.62 (95% CI – 1.04 to 2.52), and the other two groups not having statistically significantly raised levels.

Rankow and Tessaro (1998) reported the results of a survey conducted in North Carolina among the LB community between June and November 1995 [18]. Approximately 1,200 questionnaires were distributed and the sample size was 591 women. The age distribution is shown in Table 2. Six women had a history of breast cancer which would give a prevalence of breast cancer of 1.01%. The very small sample means the confidence intervals around the prevalence estimate will be wide. It seems unlikely that the outreach sampling method will have increased or decreased the likelihood that women with breast cancer will have volunteered to take part. However, the researchers suggested that more middle class white women over 40 years took part.

**Table 2:** **Age distribution in Rankow and Tessaro**[18]

**Age range**	**N**	**Percentage**
17-29	159	27.9%
30-39	188	33.0%
40-49	134	23.5%
50 and older	62	10.9%
Total n	543 (585 without breast cancer reported in paper)	(4.7% missing data)

Roberts et al. (2004) reported the results of a retrospective medical chart audit of patients attending a specialist women’s health services clinic providing healthcare to young, low-income women in urban San-Francisco and had significant outreach to the lesbian community [19]. Included were 433 lesbians and 586 heterosexuals with a mean age of 42.9 (SD 6.85, range 35–75). Five of the lesbians (1.2%) and three of the heterosexuals (0.5%) had had a breast cancer diagnosis. The logistic regression adjusted odds ratio was 1.00 (95% CI 0.21-4.80). The odds ratio was adjusted for age, ethnicity, employment status and disability status.

It was noticeable in the abstract and conclusions that mention was made of the differences in risk factors for breast cancer between lesbians and heterosexual women but not the similarity in breast cancer rates. However, it is likely that the sample was too small to demonstrate a difference in breast cancer diagnosis between the two groups and the 95% confidence intervals were wide. Also the study participants were relatively young.

Valanis et al. (2000) reported the results the Women’s Health Initiative Study on 93,311 heterosexual and non-heterosexual post-menopausal women [20]. Participants were recruited from 40 health centres around the USA aged 50–79 and were ethnically diverse. There were 90,578 heterosexuals (97.1%), 740 bisexuals (0.8%), 573 lesbians (0.6%) (categorised as lifetime lesbian – sex only with women ever, and adult lesbian – sex only with women after 45 years old) and 1420 women who had never had adult sex (1.5%). The mean ages varied between 56.7 and 64.8 with SDs between 5.6 and 7.4. The age-standardised prevalence of breast cancer was 4.9 for heterosexuals, 8.4 for bisexuals, 5.8 for lifetime lesbians, 7.0 for adult lesbians and 6.4 for asexual women. No variations around these figures were given.

The percentages of non-heterosexual women seem rather low. There were an additional 2697 women who preferred not to indicate their sexual orientation. The split between lifetime and adult lesbian seems rather arbitrary and the categorisation was based on recent sexual behaviour rather than sexual orientation.

Zaritsky and Dibble (2010) [21] reports a subset of results from a previously published paper by [23] which compared breast cancer risk factors between lesbians and their heterosexual sisters. This subset investigated the pairs where one or both sisters were aged over 50 years. There were 42 pairs (from the original study which had 370 pairs aged 40 or more) with a mean age of 63.9 (SD 8.0) for lesbians and 64.2 (SD 6.6) for the heterosexual sisters. For question about breast cancer, results were presented for 41 pairs and twelve of the lesbians (29.3%) and six of the heterosexual sisters (14.6%) had been diagnosed with breast cancer (p = 0.2).

This interesting paper is limited in population because it could only include lesbians who had a heterosexual sister. Therefore, a relatively large number of lesbians who have no siblings, only lesbian sisters or only brothers would be excluded. Also, women whose sisters had already died (possibly from breast cancer) would also not have been eligible to take part in the study. The study was also relatively small. Also, the prevalence of breast cancer found was remarkably high. Although the recruitment strategy is not clear, it seems likely that participants were recruited on the basis of being lesbians with breast cancer, or with an interest in breast cancer. Therefore, since the snowball recruitment was via the lesbian community, it is more likely that the lesbians would have had a higher rate than their heterosexual sisters.

The Prescription For Change Survey was a survey conducted on behalf of the Stonewall charity by Ruth Hunt and Dr Julie Fish, with assistance from Sigma Research (a unit that specialises in sexual health research in men who have sex with men), associated with the University of Portsmouth. It was funded by Lloyds TSB Foundation. The report [6] is available on the Sigma Research website, has no ISBN number and has never been published. The data analysis and report was written by Ruth Hunt and has not been peer reviewed. There seems to be discrepancy around the number of included women as the report mentions 6178 responses whereas a recent unpublished report by Dr Julie Fish (Coming out about breast cancer, 2009) mentions “a study of 5909 LB women”. The survey asked about demographic information, health behaviours and a wide range of physical, mental and sexual health issues and experience of healthcare provision. The specific question asks “Have you even been diagnosed with breast cancer? (NB ‘even’ should have read ‘ever’ but was evidently a misprint). The results were given in the report as:

“one in twelve lesbian and bisexual women aged between 50 and 79 have been diagnosed with breast cancer, compared with one in twenty women in general”.

The “one in twenty” statistic is not referenced so it is unclear where this has come from. The age distribution of the sample is not known, other than the range of 14–84. If a roughly normal distribution is assumed, the sample size will be approximately 2954 women. However, it is likely that the distribution will be skewed towards the younger age groups so the sample size will be less than this.

### Risk factor models and estimates

There were eleven relevant papers, which have been split into three parts – risk models, incidence model and risk factor estimates. Table 3 lists the four risk model studies in LB women compared to heterosexual women. Table 4 lists studies assessing the percentages of individual risk factors in LB women compared to heterosexual women where risk models were not used to combine results.

**Table 3:** Risk factor model results

	**Sample size, age**	**Data collection**	**Model used**	**Factors included**	**Results**
Brandenburg [15] (USA)	550 lesbian, 279 heterosexual (numbers reported don’t quite add up) (mean age 43 (SD 11))	Cross-sectional survey (possibly higher risk group)	Gail	Gail - Age, age at menarche, age at first live birth, nulliparity, previous breast biopsy, biopsy with a typical hyperplasia, first degree relative with breast cancer	5-year risk higher in lesbians (mean 0.96% lesbians, 0.85% heterosexual women, p = 0.04)
					Lifetime (to age 90) risk higher in lesbians (mean 11.6% lesbians, 10.7% heterosexual women, p = 0.001)
Bryn Austin [24] (USA)	665 lesbian, 309 bisexual, 86,418 heterosexual (age range 25–42 at baseline)	Prospective population cohort study (premenopausal, normal risk group)	Rosner-Colditz	Age, age at menarche, duration of premenopause, age at first birth, number of births, mean BMI during premenopause, height at baseline, mean alcohol intake during premenopause, history of benign breast disease, family history of breast cancer	Incidence rate ratio lesbian: heterosexual 1.06 (95% CI 1.06-1.06)
					Incidence rate ratio bisexual: heterosexual 1.10 (95% CI 1.10-1.10). One year incidence rates per 100,000 person-years were 131.6 lesbian, 131.7 bisexual and 122.6 heterosexual
Dibble [23] (USA)	324 lesbians (mean age 49.7) 324 heterosexual sisters (mean age 48.9)	Cross-sectional survey (high risk group)	Modified Gail model	Menarche age, menopause age, contraceptive pill use, parity, HRT, breastfeeding, hysterectomy, BMI, waist/hip ratio, exercise, smoking, alcohol use, low-fat diet, radiotherapy treatment	5-year risk higher in lesbians (mean 1.21% lesbians, 1.07% heterosexual women, p < 0.001)
					Lifetime risk higher in lesbians (mean 11.1% lesbians, 10.4% heterosexual women, p = 0.001)
McTiernan et al. [25] (USA)	65 lesbians (mean age 40.8 (SD 8.7)) 317 general sample (mean age 42.2 (SD 11.0))	From RCTs of breast cancer risk counselling methods (high risk group)	Gail and Claus	Gail - Age, age at menarche, age at first live birth, nulliparity, number of previous breast biopsies, biopsy with atypical hyperplasia, first degree relative with breast cancer	Current age to 79 (lifetime) risk
					Gail - lesbians 13.2 (SD 5.0), general sample 14.2 (SD 4.3)
					Claus lesbians 10.9 (SD 4.9), general sample 11.8 (SD 5.1)
				Claus – age, relative with breast cancer and their age of onset	

**Table 4:** Risk factor calculation results

	**Sample size, age**	**Data collection**	**Factors assessed**	**Results**	**Comments**
Case [27] (USA)	694 lesbian, 90129 heterosexual Age 32-51	Nurses Health Study II	Nulliparity, alcohol use	Higher prevalence of risk factors for breast cancer in lesbians	Remarkably small percentage lesbian sample of 0.8%,
Cochran [16] (USA)	11,876 lesbians, approx 19,000 women. Ages 18-75	Two national and five regional lesbian surveys, NHANES III, NHIS	Obesity, alcohol use, smoking, parity, contraceptive use, health insurance, mammography	Higher prevalence of risk factors for breast cancer in lesbians	There will be some lesbians in the comparator surveys
Grindel [26] (USA)	1139 lesbian ?n comparators	Self-administered questionnaire compared to various US national surveys	Family history of cancer, smoking, nutrition, exercise, body weight, alcohol use, HRT, sunscreen use, mammography,	Very similar prevalence of risk factors in lesbians compared to national surveys	Poor quality study, multiple comparator surveys means results difficult to interpret
Rankow [18] (USA)	591 lesbian, ?n comparators	Regional lesbian survey, BRFSS, NHANES III, NHIS,	Family history, nulliparity, overweight (BMI > 27.3), alcohol use, menarche < 12 years, mean age at menarche	“Some lesbian and bisexual women may be more likely to possess certain characteristics associated with increased breast cancer risk”	Poor quality study, multiple comparator surveys means results difficult to interpret
Roberts [19] (USA)	433 lesbian, 586 heterosexual (mean age 42.9)	Retrospective medical record audit	Alcohol use, smoking, parity, contraceptive pill use, HRT, family history, BMI, menarche age, menopause age, mammogram age,	Higher prevalence of some risk factors for breast cancer in lesbians	Likely that the sample are poorer and lower social class than a standard population
Valanis [20] (USA)	573 lesbian, 740 bisexual 90,578 heterosexual 1,420 asexual Mean ages between 56.7 and 64.8	Women’s Health Initiative survey	Mammogram, diet, smoking, alcohol use, overweight, exercise, psychosocial characteristics, contraceptive pill, HRT, pregnancy, hysterectomy,	Higher rate of engaging in risky behaviours which contribute to a higher risk for breast cancer	Small percentage lesbian/bisexual sample of 1.4%, 2.8% preferred not to indicate their sexual orientation so were excluded from analysis

### Risk models

Bryn Austin (2012) used the Rosner-Colditz model [24] and the other three studies used the Gail model [15,23,25]; one also used the Claus model [25]. The Gail model results showed conflicting estimates of risk for lesbians compared to the comparator groups. Two suggested that there was a higher risk in lesbians and the other suggested the opposite. The Rosner-Colditz model showed a higher risk in LB women.

Brandenburg et al., [15] had a relatively large lesbian sample (n = 550) but a smaller sample of heterosexual women (n = 279) collected through a community cancer project survey so may have attracted higher risk women. Also, the authors stated that they did not have data on the number of breast biopsies so all who had had a breast biopsy were assumed to have had only one biopsy. Unfortunately the numbers given for participants in the methods section does not match the explanation in the results section, which does not give confidence in the reliability of the results.

In Bryn Austin (2012) [24] prospective data from a large cohort study was used. It had the largest sample of lesbians (n = 665) and bisexual women (n = 309) which were compared to a much larger sample of heterosexual women (n = 86,418). The percentage of LB women in the sample was relatively low at 1.1% so it is likely that some LB women were included in the heterosexual sample. There were insufficient numbers of post-menopausal women to estimate risk so the analyses were restricted to pre-menopausal women and the authors noted that disparities in risk of breast cancer in post-menopausal LB women may be different to those observed in the study. The Rosner-Colditz model tends to be more accurate than the Gail model [12] so it is likely that this estimate for pre-menopausal women is relatively accurate.

In Dibble (2004) [23] the sample is limited to lesbians who had a heterosexual sister, as discussed previously. The researchers could not include atypical hyperplasia results from biopsies into the model as they had no access to these results.

In McTiernan (2001) [25] all participants had a family history of breast cancer. Recruitment was conducted slightly differently for the general sample than the lesbian sample. All recruitment specifically stated that the study was about counselling for women with a family history of breast cancer so may well have obtained a biased sample if the lesbian participants were more worried about breast cancer than the general sample. However, there was no statistically significant difference between the two groups on perceived lifetime risk of breast cancer.

### Incidence estimate

Boehmer et al., [5] tested the hypothesis that breast cancer incidence rates might be higher in geographical areas where more LB women live by linking a US cancer registry to a census. They used the following:

•Twelve registries from the Surveillance, Epidemiology and End Results (SEER) database for years 1996–2004.

•Census 2000 results for two questions (female sex of each household member and unmarried partner) which were linked to find same sex female partner households.

•Public Use Microdata Sample of the Census 2000 to estimate age and socioeconomic status of the sample.

Because they were unable to link individuals they assessed density of female same sex partnered households at a county level, and then compared the breast cancer rates. The highest density was in San Francisco and the lowest in Iowa. The results showed that a 1% increase in same sex partnered households was associated with a 13% increase in breast cancer, after adjusting for age, ethnicity and socio-economic status. However, this is an ecological study so may have ecological bias; specifically that it is unknown whether the women in the same sex partnered households are the same as the women who are getting the breast cancer.

### Risk factor estimates

All of the six studies described in Table 4 recorded a variable number of known risk factors for breast cancer. Each study looked at a slightly different list and the results were assessed and reported for each risk factor separately. Five of the six studies concluded that there was a higher risk of breast cancer based on the factors they had assessed and one concluded that it was unlikely that there would be any difference in risk between heterosexuals and lesbians.

The six studies were heterogeneous in a variety of different ways. The lesbian participants were recruited mostly via self-administered questionnaires in cross-sectional surveys but one was an audit of medical records. The comparators were mostly drawn from large US national surveys such as NHANES III and NHIS and the numbers in these national surveys actually used for comparison purposes in the studies was not stated in two of the studies. It is likely that there will have been some lesbians in the comparator group in Cochran 2001, [16,18,26] but fewer in Case 2004 [27] and Valanis 2000 [20]. This is because the first three studies compared lesbian survey results to the full national survey results whereas the second two studies had sexual orientation measured within national surveys so could compare within the survey itself. It is likely therefore, that these two latter comparisons will be more accurate. However, there was not full disclosure of sexual orientation in Case 2004 and Valanis 2000, suggested by the small percentage of lesbians in Case 2004, and that in Valanis 2000 some 2.8% of participants preferred not to give their sexual orientation. The results of Case 2004 will have been diluted by some lesbians being included within the heterosexual group whereas in Valanis 2000, the “prefer not to say” group were excluded.

Therefore, of the six studies, it is likely that Valanis 2000 may have more believable results than the others. However, all of the studies are from the USA and it is unclear how generalisable these results are to LB women in other countries because of differences in modifiable risk factors such as obesity rates and use of alcohol in different countries.

## Discussion

This systematic review found no incidence estimates for breast cancer in individual LB women. There were twenty included studies using a variety of study designs to estimate prevalence, risk and local population incidence rates. This is a considerable body of research and suggests that there much interest in the question as to whether there is a higher incidence of breast cancer in lesbians and bisexual women than heterosexual women.

Of the prevalence estimates, all of the studies found were relatively small and had a variety of poor quality issues associated with study design or reporting. The one unpublished estimate was high at 8% [6], relative to an estimated UK 34-year prevalence of 1.5% [28]. In the published estimates, one showed higher prevalence [20], four showed no difference between heterosexual and LB women [16,21,22,29], one showed mixed results depending on definitions [17], one had no comparison group [30] and one gave no sample size [15].

Four risk modelling studies, one population incidence model and six risk factor estimates came from the USA. In two of the modelling studies, the populations may have been either high risk of breast cancer because of family history or at higher risk because of sampling biases. Three of the risk modelling studies suggested a higher rate of breast cancer in LB women compared to heterosexual women and one gave the opposite. The local population incidence rate model suggested a higher incidence of breast cancer in regions where more same sex partnered households live. Of the six risk factor estimate studies in LB women, five of the six studies concluded that there was a higher risk of breast cancer based on the factors they had assessed and one concluded that it was unlikely that there would be any difference in risk.

### Strengths and limitations

A strength of this project is the comprehensiveness of the systematic review, which includes unpublished as well as published evidence in order to minimise publication bias. It has been conducted by very experienced systematic reviewers. The searches were updated to very close to the publication date to ensure no studies were missed.

There are limitations from the conduct of the review and the weakness of the included studies. Coding of prevalence studies and the other types of studies included in this systematic review in medical databases is poor so there is a greater risk of missing relevant studies that could have been included, compared to systematic reviews of, for example, randomised controlled trials. Systematic reviews of prediction studies are rare [31] and there is no commonly accepted template. There are no generally accepted or validated quality criteria for assessment of prevalence studies and modelling studies available so quality assessment has been based on pragmatic criteria adapted from other publications. A considerable limitation is the lack of good quality information on breast cancer incidence and prevalence and known breast cancer risk factors in LB women.

Risk would need to be assessed prospectively in order to ensure that the risk factor occurred before the development of disease and did not start after diagnosis. This is more important for some potential risk factors such as alcohol use. There is an assumption that if a certain risk factor is present, then acting on that risk factor, such as drinking less alcohol, will substantially alter the risk of breast cancer. For some of these risk factors, it is unclear whether the factor itself is linked with incidence, or whether the factor is correlated with another causative factor. However, losing weight after the menopause is associated with less risk because of the oestrogenic effects of adipose tissue.

## Conclusion

Given the available evidence, it remains uncertain as to whether there is a higher incidence of breast cancer in LB women or not, but the balance of evidence is starting to suggest a higher incidence. The only realistic way to establish rates in LB women compared to heterosexual women would be to collect sexual orientation within routine statistics, including Cancer Registry data or from large cohort studies. Recently the UK National Cancer Data Set was reviewed [32] but it is unclear as to whether sexual orientation will be included in the new dataset. Work by the UK Government Office for National Statistics has developed a valid and reliable measurement tool for collecting sexual orientation within interview and questionnaire-based research [33]. So far there has been little uptake in UK Government-funded research. Measurement of sexual orientation within research conducted in other countries remains patchy and so far little has been published. However, if sexual orientation is measured, surprising results may be found. For example, the recent finding that LB women have a three times higher risk of dying from breast cancer than heterosexual women came from a US Government funded survey. It is vital that sexual orientation be measured in order that health inequalities such as these can be found and addressed.

## Abbreviations

BMI: Body mass index; BRFSS: Behavioural risk factor surveillance system; CAB Abstracts: Commonwealth agricultural bureau abstracts database; CDSR: Cochrane database of systematic reviews; CENTRAL: Cochrane database of trials; CI: Confidence interval; DARE: Database of reviews of effects; EMBASE: Biomedical answers database; HRT: Hormone replacement therapy; HTA: Health technology assessment database; ISBN: International standard book number; LB: Lesbian and bisexual; LGB: Lesbian, gay and bisexual; MEDLINE: Medical database; NHANES: National health and nutrition examination survey; NHIS: National health interview survey; NHSEED: National health service economic evaluation database; PsychINFO: Psychological information database; SCI: Science citation index; SD: Standard deviation; SIGLE: System for intervention on grey literature in Europe; SSCI: Social science citation index; TSB: Trustee Savings Bank; UK: United Kingdom; USA: United States of America.

## Competing interests

The authors declare that they have no competing interests.

## Authors’ contributions

CM devised, conducted and wrote the original systematic review as her Master’s Degree in Public Health Dissertation. This dissertation was awarded the Thomas McKeown prize for the best MPH project. DM was second and senior reviewer for the subsequent publication. Both authors read and approved the final manuscript.

## Authors’ information

CM degrees - MBChB, MSc, PhD, MPH, FHEA.

DM degrees - BSc, PhD.

CM - Reader in Health Technology Assessment, Health Economics Research Group, Brunel University, Middlesex,

DM – Senior Lecturer, Unit of Public Health, Epidemiology and Biostatistics, University of Birmingham.

## Pre-publication history

The pre-publication history for this paper can be accessed here:

http://www.biomedcentral.com/1471-2458/13/1127/prepub
